# Prosthetic Breast Reconstruction and Red Breast Syndrome: Demystification and a Review of the Literature

**DOI:** 10.1097/GOX.0000000000002108

**Published:** 2019-05-23

**Authors:** Maurice Y. Nahabedian

**Affiliations:** From the Virginia Commonwealth University, Inova Branch, National Center for Plastic Surgery, McLean, Va.

## Abstract

Red breast syndrome (RBS) represents an inflammatory condition that rarely occurs in the setting of acellular dermal matrix use after prosthetic reconstruction. It is characterized by erythema or rubor that occurs directly over the ADM, and its appearance resembles that of a cellulitis. There have been many explanations with regard to etiology, but none have addressed the physiologic alterations leading to the onset and resolution of RBS. RBS is postulated to be the result of lymphatic disruption and is self-limiting. Resolution is postulated to be the result of angiolymphatic regeneration and the re-establishment of lymphatic flow within the mastectomy skin flap and the ADM, resulting in the clearance of inflammatory mediators responsible for the localized erythema.

## Abstract 2

Supplemental Digital Content is available in the text.

## INTRODUCTION

The controversy surrounding red breast syndrome (RBS) associated with acellular dermal matrix (ADM) in the setting of prosthetic breast reconstruction has been a source of controversy, confusion, and discussion.^1^ The primary stigma of RBS is cutaneous erythema directly over the territory of the ADM with an incidence ranging from 0% to 27% (Table 1).^2–10^ The controversy is focused on what it represents and why it occurs. The confusion arises at the initial presentation based on whether this represents an infection or a benign inflammatory condition. Questions, such as are there predisposing factors that may lead to RBS, why does not it occur more often, and is it a random occurrence that can occur in anyone, remain.

**Table 1.**
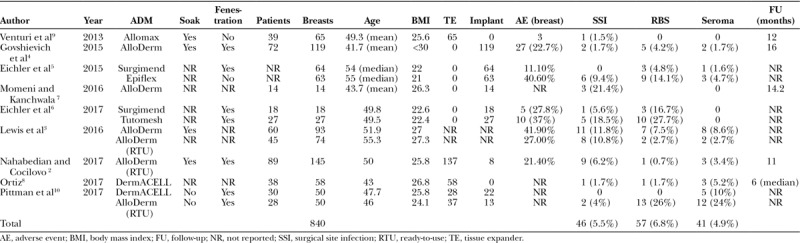
Clinical Outcomes from 9 Studies Comparing the Incidence of SSI, RBS, and Seroma

The etiology of RBS has remained elusive and has been linked to various factors. The first published article that described this phenomenon was in 2009, and the etiology was suspected to be an allergic reaction to the additives in the packaging.^11^ Replies and explanations have led to more clarity, but an exact cause has remained elusive.^12,13^ Since then, several explanations have emerged that include the orientation of the ADM (dermal versus basement membrane adjacent to the skin flap), free fat between the ADM and the skin flap, residual DNA within the ADM, neovascularization of the ADM, delayed hypersensitivity reaction to ADM, processing of the ADM, degree of ADM sterility, body mass index, and lymphatic obstruction (Table 2).^1,3,8,10,12,14^

**Table 2.**
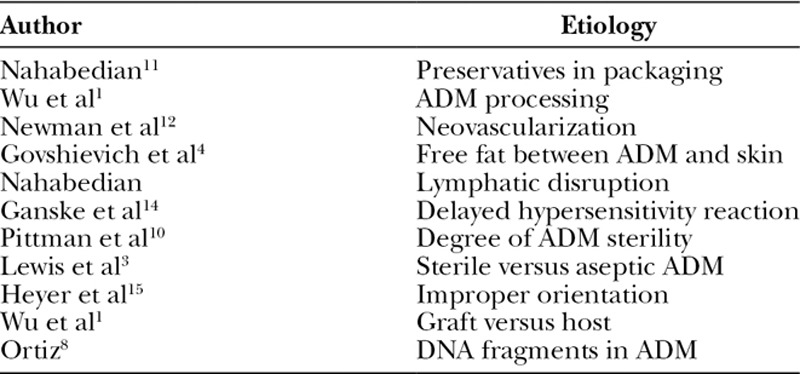
Various Etiologies of Red Breast Syndrome With the Corresponding Study.

The hypothesis of this study is that RBS is primarily due to the lymphedema and rubor of the mastectomy skin flaps that occasionally occurs in the setting of ADM. The evidence to support this hypothesis is derived from various assumptions and facts related to the vascular and lymphatic anatomy and physiology of the breast. Although the other factors may be associated with RBS, none actually provides a pathophysiologic explanation. Table 3 highlights some of the truths and myths about RBS that are important to appreciate in an attempt to understand the pathophysiology of this condition. To better understand the etiology of RBS, it is important to review the relevant anatomy and physiology of the breast.

**Table 3.**
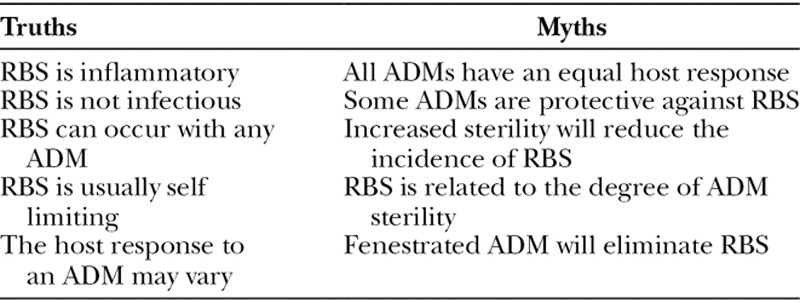
Truths and Myths of Red Breast Syndrome

## LYMPHATIC ANATOMY OF THE BREAST

A clear understanding of the normal anatomy and physiology of the breast as it relates to the lymphatic system is important.^16,17^ The breast is an ectodermal structure with a lymphatic network that parallels the skin. From the skin, lymph flow is directed to the subcutaneous plexus between the skin and the superficial fascia. The superficial lymphatic network extends from the subdermis to the deep fascia of the breast and surrounds the lobular units of the breast. The lymphatic plexus of each of breast lobule merges to form the Sappey subareolar plexus. The superficial breast lymphatics converge into the Sappey plexus that is connected to the deep fascial plexus through fibrous strands traversing the breast. Lymphatics often parallel the vascular network. The subdermal vascular network is in continuity with the superficial and deep system of vessels.

The nature of the superficial lymphatic drainage of the breast is exemplified by appreciating the technique of sentinel lymph node biopsy.^18^ After the injection of dye in the periareolar dermis, absorption occurs through the lymphatic capillaries that range in diameter from 20 to 70 µm.^17^ From the capillaries, lymph is directed to the lymphatic precollectors ranging from 70 to 150 µm. These lymphatic networks are located in the dermis between the reticular and papillary layers. From the precollectors, the dye is directed to the superficial lymphatics located in the subcutaneous tissue and range in diameter from 150 to 350 µm. The superficial lymphatic system drains into the deep lymphatic system located beneath the deep fascia of the breast and ultimately to the regional lymph nodes.

## POSTMASTECTOMY LYMPHATICS AND VASCULARITY

After mastectomy, the breast parenchyma is excised with moderate-to-severe disruption of the vascular and lymphatic networks that can have a significant impact on the residual vascularity and lymphatic drainage of the mastectomy skin flap. In addition, the absorptive capacity of the mastectomy skin flaps is compromised and the transport of lymphatic fluid is disrupted. The thickness of the subcutaneous fat and the surface dimensions of the remaining mastectomy skin can impact lymphatic flow, drainage, and function. The superficial lymphatics that are located within the subcutaneous layer are most susceptible to injury based on the boundaries of the mastectomy. When a mastectomy is performed without reconstruction, the skin flaps are placed on the chest wall in contact with the pectoralis major muscle. Assuming that the perfusion to the skin flaps is sufficient, normal wound healing will occur and allow for the re-establishment of vascular and lymphatic connections over time. In this setting, seromas that occur are usually due to excessive fluid production or shear between the surfaces disrupting the normal contact healing that occurs.

## PROSTHETIC RECONSTRUCTION WITHOUT ADM

In the setting of prosthetic reconstruction without an ADM or with autologous reconstruction, the mastectomy skin flaps will be in contact with the prosthetic device, pectoralis major muscle, or the soft tissues of the flap. Although the lymphatic drainage of the mastectomy skin flaps is initially disrupted, it is not impeded, as there is no obstructive barrier placed along the cut lymphatic vessels. Placement of a breast implant or tissue expander will not cause entrapment of lymphatic fluid because the lymphatic fluid will drain toward the chest wall and be absorbed or contribute to seroma formation. Normal wound healing will allow for angiogenesis and lymphangiogenesis within the mastectomy skin flaps to occur over time. However, if lymphangiogenesis fails to occur or there is obstruction of lymphatic drainage system, the occurrence of lymphedema is likely and is typically manifest by rubor and swelling. It is accepted that some degree of postoperative edema of the mastectomy skin flaps is normal resulting from tissue trauma. When lymphatic obstruction is protracted, rubor and possibly pitting edema of the mastectomy skin can be observed but will usually resolve once lymphangiogenesis has been initiated. This cutaneous rubor is also occasionally seen in women having reduction mammaplasty and is due to lymphatic dysfunction.^19^ Studies have demonstrated a normal return of breast lymphatic drainage after reduction mammaplasty.^20^

## PROSTHETIC RECONSTRUCTION WITH ADM

**Fig. 1.**
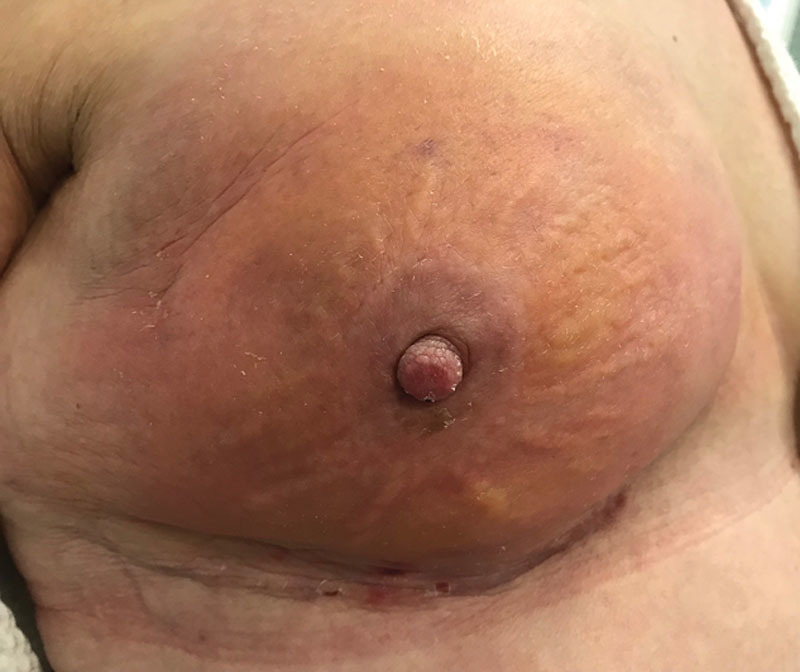
Red breast syndrome in a patient after prepectoral breast reconstruction with ADM.

In situations where an ADM is used, the fate of the disrupted lymphatics in the subcutaneous fat becomes less clear. It is important to appreciate that the interface between ADM and a mastectomy skin flap is very different than that of an implant and mastectomy skin flap because the lymphatic fluid is not trapped in the setting of an implant alone; it is either removed by the drains or drains into the periprosthetic space and is absorbed by the surrounding soft tissues/chest wall. The divided superficial lymphatic vessels that would normally traverse through the subcutaneous fat toward the deep lymphatics in the normal breast or toward the pectoral muscle or autologous fat after traditional reconstruction are now in direct contact with the freshly placed ADM that may in some cases create an obstructive barrier to lymphatic drainage or leakage, resulting in the entrapment of lymphatic fluid, lymphedema, and rubor. This will typically persist until angiogenesis and lymphangiogenesis occur, primarily within the mastectomy skin flaps and secondarily within the ADM. The angiogenic and lymphangiogenic potentials of the ADM are important considerations in this setting. When RBS is present, the cutaneous erythema is usually localized to that territory overlying the ADM (Fig. 1). Lymphedema of the soft tissues can also occur in more advanced cases (**see** video, Supplemental Digital Content 1, which displays pitting edema demonstrated in a patient with red breast syndrome, http://links.lww.com/PRSGO/B78)

**Video Graphic 1.**
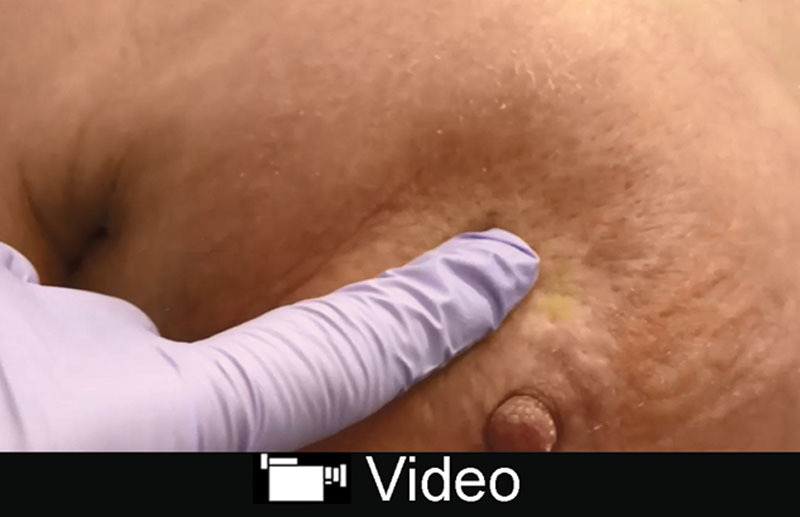
See video, Supplemental Digital Content 1, which displays pitting edema demonstrated in a patient with red breast syndrome, http://links.lww.com/PRSGO/B78.

The orientation of the ADM within the mastectomy space is important because human ADM has polarity with a dermal and a basement membrane surface. It is a commonly accepted strategy to place the dermal side of the ADM toward the mastectomy skin, because this will increase the likelihood of revascularization, recellularization, and incorporation.^15,21^ The use of closed suction drains placed internally or the application of an negative pressure incisional therapy device will create negative pressure internally and facilitate direct contact between the ADM and the mastectomy skin flap and promote early incorporation.^22^

The formation of a seroma is the most common adverse event in the setting of an ADM with an incidence that ranges from 5% to 12% based on studies that did not mention RBS^22–25^ and an incidence ranging from 0% to 24% with a mean of 4.9% based on studies that did mention RBS (Table 1). Seromas can be clinically evident and manifest as a fluid wave or subclinically without any external evidence. A seroma can occur between the ADM and the device and between the ADM and skin flap. Seromas between the skin and ADM will impede the revascularization and recellularization of the ADM and thus impede angiogenesis within the ADM but should not impede angiogenesis and lymphangiogenesis within the mastectomy skin flap. Seromas in this location are also characterized by the gradual formation of a thin capsule that forms over the subcutaneous fat that may impede lymphatic flow resulting in edema and rubor. The use of perforated or fenestrated ADM has been postulated to allow for egress of fluid between the skin and ADM and, thus, decrease the incidence of early seroma formation.^26–29^

## EVIDENCE FOR ADM REVASCULARIZATION

**Fig. 2.**
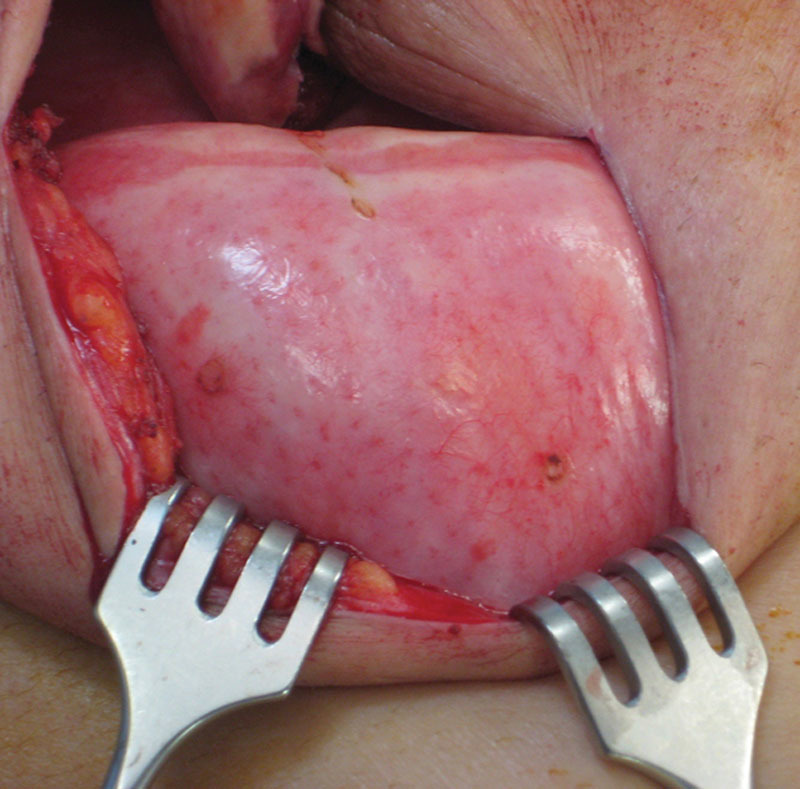
A pink hue, vascularized follicles and the demonstration of small vascular channels characterize revascularized ADM.

It is important to recognize that unlike a skin graft where inosculation occurs between days 2 and 5, the revascularization of an ADM has a more protracted course. The evidence for this is derived from experimental studies looking specifically at this. In a murine study evaluating ADM revascularization, it was demonstrated that increased oxygen consumption and angiogenesis along the edges of the ADM occur from days 10 to 14 with vascular and inflammatory cell penetration into the center of the ADM after about 21 days.^30^ In another experimental study using a porcine model, it was demonstrated that early angiogenesis occurs at 4 weeks at the interface of the ADM and skin flap.^31^ The revascularization of both surfaces of the ADM is evident by 8 weeks (Fig. 2). Microcirculatory evaluation using a video microscope demonstrated detectable flow 12 weeks after implantation. These histological changes related to angiogenesis may serve as a foundation for the self-limiting nature of RBS.

## ADM ADHERENCE VERSUS REVASCULARIZATION

It is important to differentiate ADM adherence from revascularization, because the two are distinctly different. Adherence alone, due to scar formation, does not imply revascularization or recellularization and will not provide any physiologic function or benefit. Clinical evidence for revascularization is based on a pink hue, vascularized hair follicles, and actual vascular ingrowth (Fig. 2). In order for lymphatic function within ADM to occur, revascularization and recellularization of ADM are required. Lymphatic function will be compromised in the presence of dense scar; therefore, it is important to use an ADM that will confer regenerative potential rather than a scarred scaffold. It is important for clinicians to be aware of the differences in ADM performance and select accordingly.

## LYMPHATIC OBSTRUCTION AND RBS

When the normal path of lymphatic flow is obstructed or the mechanical forces promoting lymphatic flow are disrupted, erythema and edema of the mastectomy skin flaps may ensue.^32^ This may be the result of various inflammatory mediators that in some cases may be associated with mild bacterial overgrowth.^32^ Early resolution of RBS is most likely the result of lymphangiogenesis within the mastectomy skin flaps and ADM that will gradually result in a diminution of the erythema. This process may be expedited by ADMs that revascularize and recellularize relatively quickly. Late resolution of or persistent RBS is postulated to be due to the absence of lymphatic connections within the mastectomy skin flap and may be amplified by an ADM that has failed to revascularize or recellularize, thus never permitting the normal drainage of the cutaneous lymphatic system.

## INFECTION VERSUS RBS

**Fig. 3.**
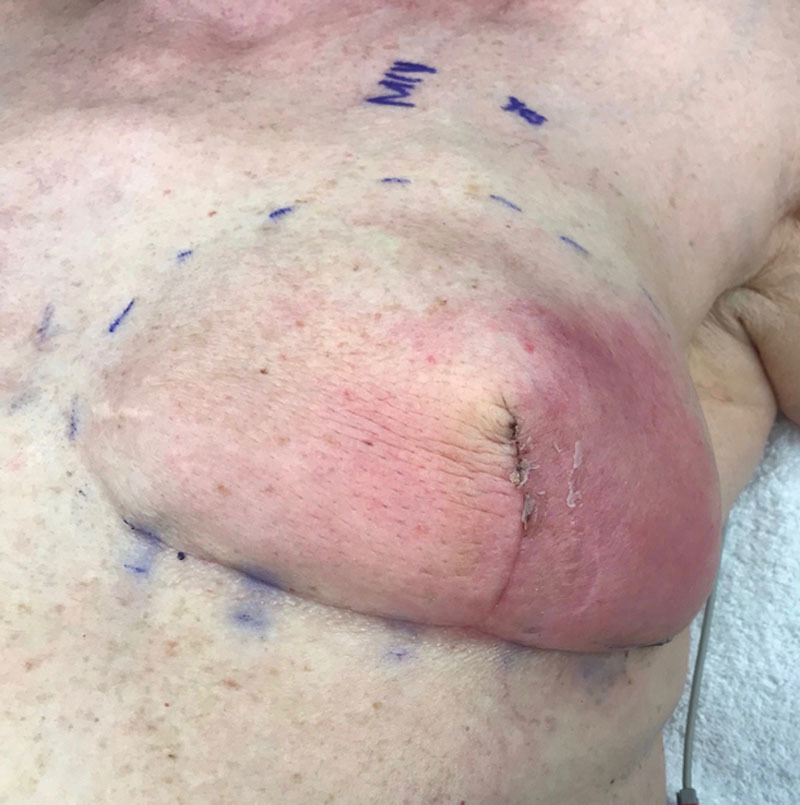
Red breast syndrome can mimic the appearance of cellulitis

**Fig. 4.**
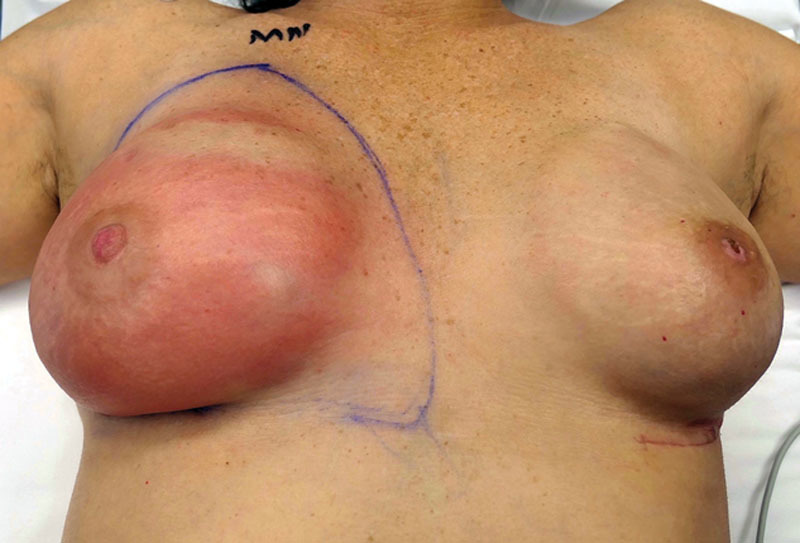
Cellulitis can mimic the appearance of red breast syndrome.

**Fig. 5.**
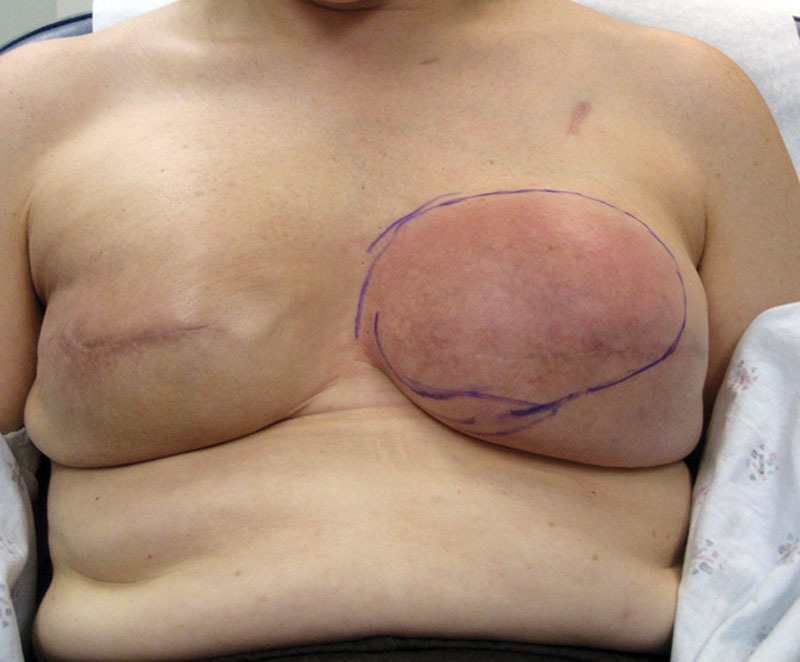
An example of erythema in a patient with dual plane breast reconstruction. The redness extends outside the borders of the ADM characteristic of cellulitis.

**Fig. 6.**
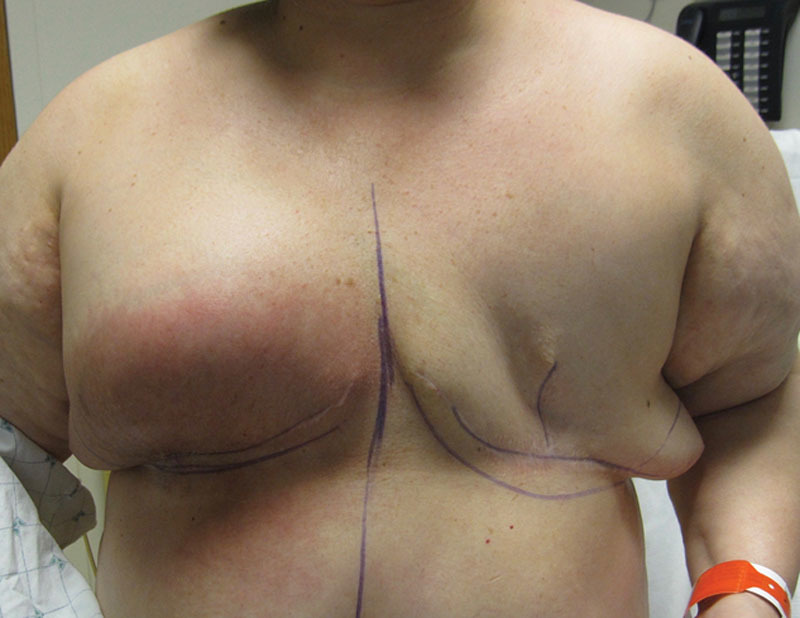
After bilateral breast reconstruction with tissue expanders and ADM. The left implant was removed secondary to infection. The right breast was erythematous for 9 months with protracted inflammation due to red breast syndrome.

The differentiation between RBS and infection is important, because both can occur within a similar timeframe and have similar clinical characteristics; however, the two have a distinctly different pathophysiology.^1,3^ RBS is usually self-limiting and will resolve without treatment, whereas infection is usually progressive and will cause deterioration and reconstructive failure over time if untreated (Figs. 3, 4). External cues that can assist in differentiating the two include the extent or location of the erythema. The erythema associated with RBS is usually over the ADM, whereas the erythema associated with infection may extend beyond the borders of the ADM. When the erythema extends outside the territory of the ADM, superficial or deep infection must be considered (Fig. 5). In some cases of protracted RBS, the erythema can extend beyond the borders of the ADM (Fig. 6).

**Fig. 7.**
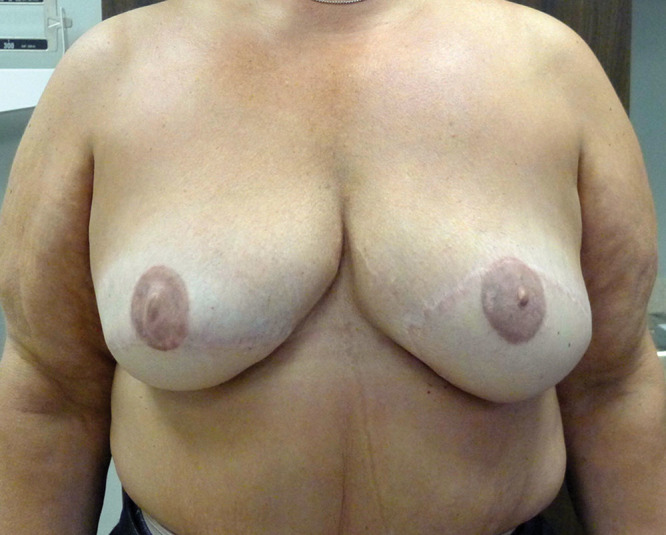
After explantation and bilateral breast reconstruction with latissimus dorsi musculocutaneous flaps and implants.

When breast erythema is noted after mastectomy and ADM use, the initial response should be to rule out infection. The incidence of surgical site infection in the setting of ADM ranged from 0% to 18.5% with a mean incidence of 5.5% in the 9 studies reviewed (Table 1). The initial evaluation requires a through history and physical examination and obtaining appropriate laboratory studies. Prior investigation has demonstrated that the primary hallmarks of infection are pain, redness, and swelling and that fever and leukocytosis are less common.^33^ In patients with RBS, fever, leukocytosis, and pain are uncommon; however, erythema is universal with or without breast pain or edema. The onset and duration of RBS is variable ranging from a few days to a few weeks and a few weeks to several months, respectively. The self-limiting aspect of RBS is postulated to be the result of angiolymphatic regeneration and the re-establishment of lymphatic flow, thereby resulting in the resolution of the inflammatory mediators responsible for the localized erythema.

In all cases of erythema, a trial of antibiotic therapy is recommended that may be administered orally if mild or intravenously if severe. Cellulitis will typically resolve with antibiotics or require operative exploration if progressive. RBS will usually be unaffected with antibiotic therapy but can progress to infection if there is a component of bacterial overgrowth. If there is no change after 1 week of therapy and the patient remains afebrile, RBS is presumed and the antibiotics are discontinued. Figures 6, 7 illustrate a patient with RBS of 9 months duration who eventually had explantation of the original implants and ADM followed by secondary reconstruction. The decision to convert from prosthetic to autologous reconstruction in the setting of protracted RBS is based on the quality of the reconstruction, patient concerns, and surgeon recommendation.

## UNDERSTANDING ADM STERILITY

Several retrospective clinical studies have reviewed the incidence of RBS and attempted to determine its etiology without any physiologic explanations (Table 1). In one study comparing aseptic AlloDerm to sterile AlloDerm, it was demonstrated that the incidence of RBS decreased from 7.5% to 2.5%.^3^ In another study, the incidence of surgical site infection was 11.1% with aseptic and 7.7% with sterile AlloDerm.^24^ The difference between aseptic and sterile AlloDerm is that the aseptic AlloDerm is freeze dried and has a sterility assurance level (SAL) of 10^−3^, whereas sterile AlloDerm is terminally sterilized using radiation and has an SAL that is also 10^−3^. Given that the incidence of RBS was reduced but not eliminated with the sterile product, the authors recognized that the occurrence of RBS might be unrelated to the processing of ADM. Although other ADMs were not evaluated, the study implied that RBS could occur with any ADM. In another study using a mathematical model to evaluate the relationship of SAL to infection, it was demonstrated that there was no difference in the rate of infection when comparing ADM with an SAL of 10^−3^ and 10^−6^.^34^

The purpose of device or tissue sterilization is to reduce the bacterial count. Guidelines for sterilization set forth by the Food and Drug Administration (FDA) are that for a product to be considered sterile, a minimal SAL of 10^−3^ must be achieved using terminal sterilization techniques such as radiation and detergents. An SAL of 10^−3^ implies that the likelihood of finding a viable organism is one in a thousand, whereas an SAL of 10^−6^ would be one in a million. Standards for the sterilization of medical devices or tissues will depend on the nature of the material. Materials that are heat resistant such as metals are best sterilized to an SAL ranging from 10^−6^ to 10^−9^. This is in contrast to materials that are heat sensitive that are typically sterilized to an SAL of 10^−3^.^35^ Human acellular dermal matrices are thermally sensitive tissues that can be damaged by excessive radiation. The implantation of a damaged human ADM is far more likely to result in an inflammatory reaction as the body undergoes degradation processes to eradicate the material from the body. It is postulated that the refractory nature of the RBS may be the result of scarred interface between the ADM and the mastectomy skin flap compromising the flow of lymphatic fluid and resulting in protracted RBS.

## CLINICAL STUDIES

There have been several comparative clinical outcome studies evaluating various ADM materials. In 1 publication comparing dual plane reconstruction using AlloDerm (Allergan Inc., Irvine, CA) to DermACELL (Stryker, Kalamazoo, MI), the authors concluded that RBS was increased with AlloDerm (26%) compared with DermACELL (0%).^10^ The primary explanation for this observation was that DermACELL was sterilized to an SAL of 10^−6^, whereas AlloDerm was sterilized to an SAL of 10^−3^. The authors concluded that RBS is an inflammatory response to ADM and that by aggressive sterilization of ADM, RBS would be eliminated. Their conclusion that RBS is due to the degree of ADM sterilization is not based on any physiologic explanation and represents conjecture. Their contention that RBS is inflammatory is accurate; however, the implication that it is minimized by increasing the SAL to 10^−6^ is without foundation and misrepresentative.

It is important to recognize that RBS is not product specific. It can occur with any ADM regardless of the degree of sterilization or the biologic source (Table 1). This has been demonstrated by the clinical studies and personal observation having used a variety of ADMs and having evaluated patients with RBS who have had different ADMs placed (Table 1). It is also important to appreciate that RBS is uncommon with a mean occurrence based on review of the 8 studies of 6.4%.

## CONCLUSIONS

In conclusion, RBS is more likely to represent the rubor associated with lymphedema and lymphatic obstruction rather than the type of ADM used or the other possible etiologies mentioned. It is important to recognize that these conclusions are based on the best available evidence and is not intended to be absolute. Inflammation is multifactorial, but the clinical appearance and characteristics of RBS are constant. Understanding the possible mechanisms responsible for RBS is important, as we move forward with prosthetic breast reconstruction and ADM.

## Supplementary Material
