# Supplementary material for: Prosthetic Breast Reconstruction and Red Breast Syndrome: Demystification and a Review of the Literature
Source: Plast Reconstr Surg Glob Open. 2019 May 23;7(5):e2108. doi: 10.1097/GOX.0000000000002108 (PMC6571326; doi:10.1097/GOX.0000000000002108)
Supplement: Supplementary file 1 [file gox-7-e2108-s001.docx]

Prosthetic Breast Reconstruction and Red Breast Syndrome: Demystification and a Review of the Literature

Maurice Y. Nahabedian, MD

McLean, VA

Correspondence:

Maurice Y. Nahabedian, MD

Professor of Plastic Surgery

Virginia Commonwealth University – Inova Branch

National Center for Plastic Surgery

7601 Lewinsville Dr., #400

McLean, VA 22102

Phone: 301-2752297

Email: DrNahabedian@aol.com

Key Words: Red breast syndrome, acellular dermal matrix, breast implants, tissue expanders, AlloDerm, prepectoral, breast reconstruction, silicone gel implant

Dr Nahabedian is Chief Surgical Officer for PolarityTE (Salt Lake City, UT) and a consultant for Allergan (Irvine, CA). No funding or support was obtained in the preparation of this manuscript

**Abstract**

Red breast syndrome represents an inflammatory condition that rarely occurs in the setting of acellular dermal matrix use following prosthetic reconstruction. It is characterized by erythema or rubor that occurs directly over the ADM and its appearance resembles that of a cellulitis. There have been many explanations with regard to etiology but none have addressed the physiologic alterations leading to the onset and resolution of red breast syndrome. Red breast syndrome is postulated to be the result of lymphatic disruption and is self-limiting. Resolution is postulated to be the result of angiolymphatic regeneration and the re-establishment of lymphatic flow within the mastectomy skin flap and the ADM resulting in the clearance of inflammatory mediators responsible for the localized erythema.

**Introduction**

The controversy surrounding red breast syndrome (RBS) associated with acellular dermal matrix (ADM) in the setting of prosthetic breast reconstruction has been a source of controversy, confusion and discussion (1). The primary stigma of RBS is cutaneous erythema directly over the territory of the ADM with an incidence ranging from 0 - 27%. (2-10) (Table 1) The controversy is focused on what it represents and why it occurs. The confusion arises at the initial presentation based on whether this represents an infection or a benign inflammatory condition. Questions such as, are there predisposing factors that may lead to RBS, why doesn’t it occur more often and is it a random occurrence that can occur in anyone, remain.

The etiology of RBS has remained elusive and has been linked to various factors. The first published manuscript that described this phenomenon was in 2009 and the etiology was suspected to be an allergic reaction to the additives in the packaging (11). Replies and explanations have led to more clarity but an exact cause has remained elusive (12,13) Since then, several explanations have emerged that include the orientation of the ADM (dermal vs basement membrane adjacent to the skin flap), free fat between the ADM and the skin flap, residual DNA within the ADM, neovascularization of the ADM, delayed hypersensitivity reaction to ADM, processing of the ADM, degree of ADM sterility, body mass index (BMI), as well as lymphatic obstruction (1,3,8,10,12,14) (Table 2)

The hypothesis of this study is that RBS is primarily due to lymphedema and rubor of the mastectomy skin flaps that occasionally occurs in the setting of ADM. The evidence to support this hypothesis is derived from various assumptions and facts related to the vascular and lymphatic anatomy and physiology of the breast. Although the other factors may be associated with RBS, none actually provides a pathophysiologic explanation. Table 3 highlights some of the truths and myths about RBS that are important to appreciate in an attempt to understand the pathophysiology of this condition. In order to better understand the etiology of RBS, it is important to review the relevant anatomy and physiology of the breast.

**Lymphatic Anatomy of the Breast**

A clear understanding of the normal anatomy and physiology of the breast as it relates to the lymphatic system is important (15,16). The [breast](https://radiopaedia.org/articles/breast)is an ectodermal structure with a lymphatic network that parallels the skin. From the skin, lymph flow is directed to the subcutaneous plexus between the skin and the superficial fascia. The superficial lymphatic network extends from the sub dermis to the deep fascia of the breast and surrounds the lobular units of the breast. The lymphatic plexus of each of breast lobule merges to form the Sappey subareolar plexus. The superficial breast lymphatics converge into the Sappey plexus that is connected to the deep fascial plexus through fibrous strands traversing the breast. Lymphatics often parallel the vascular network. The subdermal vascular network is in continuity with the superficial and deep system of vessels.

The nature of the superficial lymphatic drainage of the breast is exemplified by appreciating the technique of sentinel lymph node biopsy (17). Following the injection of dye in the periareolar dermis, absorption occurs through the lymphatic capillaries that range in diameter from 20-70 microns (16). From the capillaries, lymph is directed to the lymphatic precollectors ranging from 70-150 microns. These lymphatic networks are located in the dermis between the reticular and papillary layers. From the precollectors, the dye is directed to the superficial lymphatics located in the subcutaneous tissue and range in diameter from 150-350 microns. The superficial lymphatic system drains into the deep lymphatic system located beneath the deep fascia of the breast and ultimately to the regional lymph nodes.

**Post Mastectomy Lymphatics and Vascularity**

Following mastectomy, the breast parenchyma is excised with moderate to severe disruption of the vascular and lymphatic networks that can have a significant impact on the residual vascularity and lymphatic drainage of the mastectomy skin flap. In addition, the absorptive capacity of the mastectomy skin flaps is compromised and the transport of lymphatic fluid is disrupted. The thickness of the subcutaneous fat and the surface dimensions of the remaining mastectomy skin can impact lymphatic flow, drainage and function. The superficial lymphatics that are located within the subcutaneous layer are most susceptible to injury based on the boundaries of the mastectomy. When a mastectomy is performed without reconstruction, the skin flaps are placed on the chest wall in contact with the pectoralis major muscle. Assuming that the perfusion to the skin flaps is sufficient, normal wound healing will occur and allow for the re-establishment of vascular and lymphatic connections over time. In this setting, seromas that occur are usually due to excessive fluid production or shear between the surfaces disrupting the normal contact healing that occurs.

**Prosthetic Reconstruction Without ADM**

In the setting of prosthetic reconstruction without an ADM or with autologous reconstruction, the mastectomy skin flaps will be in contact with the prosthetic device, pectoralis major muscle or the soft tissues of the flap. Although the lymphatic drainage of the mastectomy skin flaps is initially disrupted, it is not impeded, as there is no obstructive barrier placed along the cut lymphatic vessels. Placement of a breast implant or tissue expander will not cause entrapment of lymphatic fluid because the lymphatic fluid will drain towards the chest wall and be absorbed or contribute to seroma formation. Normal wound healing will allow for angiogenesis and lymphangiogenesis within the mastectomy skin flaps to occur over time. However, if lymphangiogenesis fails to occur or there is obstruction of lymphatic drainage system, the occurrence of lymphedema is likely and is typically manifest by rubor and swelling. It is accepted that some degree of postoperative edema of the mastectomy skin flaps is normal resulting from tissue trauma. When lymphatic obstruction is protracted, rubor and possibly pitting edema of the mastectomy skin can be observed but will usually resolve once lymphangiogenesis has been initiated. This cutaneous rubor is also occasionally seen in women having reduction mammaplasty and is due to lymphatic dysfunction (18). Studies have demonstrated a normal return of breast lymphatic drainage following reduction mammaplasty (19).

**Prosthetic Reconstruction With ADM**

In situations where an ADM is used, the fate of the disrupted lymphatics in the subcutaneous fat becomes less clear. It is important to appreciate that the interface between ADM and a mastectomy skin flap is very different than that of an implant and mastectomy skin flap because the lymphatic fluid is not trapped in the setting of an implant alone; it is either removed by the drains or it drains into the periprosthetic space and is absorbed by the surrounding soft tissues / chest wall. The divided superficial lymphatic vessels that would normally traverse through the subcutaneous fat towards the deep lymphatics in the normal breast or towards the pectoral muscle or autologous fat following traditional reconstruction are now in direct contact with the freshly placed ADM that may in some cases create an obstructive barrier to lymphatic drainage or leakage resulting in the entrapment of lymphatic fluid, lymphedema and rubor. This will typically persist until angiogenesis and lymphangiogenesis occurs, primarily within the mastectomy skin flaps and secondarily within the ADM. The angiogenic and lymphangiogenic potential of the ADM are important considerations in this setting. When RBS is present, the cutaneous erythema is usually localized to that territory overlying the ADM (figure 1). Lymphedema of the soft tissues can also occur in more advanced cases (SDC1) (See video, Supplemental Digital Content 1 which displays pitting edema demonstrated in a patient with red breast syndrome. http://links.lww.com/PRSGO/B78)

The orientation of the ADM within the mastectomy space is important because human ADM has polarity with a dermal and a basement membrane surface. It is a commonly accepted strategy to place the dermal side of the ADM towards the mastectomy skin because this will increase the likelihood of revascularization, recellularization and incorporation (20,21). The use of closed suction drains placed internally or the application of an negative pressure incisional therapy device will create negative pressure internally and facilitate direct contact between the ADM and the mastectomy skin flap and promote early incorporation (22).

The formation of a seroma is the most common adverse event in the setting of an ADM with an incidence that ranges from 5-12% based on studies that did not mention RBS (22-25) and an incidence ranging from 0 – 24% with a mean of 4.9% based on studies that did mention RBS (table 1). Seromas can be clinically evident and manifest as a fluid wave or sub-clinically without any external evidence. A seroma can occur between the ADM and the device as well as between the ADM and skin flap. Seromas between the skin and ADM will impede the revascularization and recellularization of the ADM and thus impede angiogenesis within the ADM but should not impede angiogenesis and lymphangiogenesis within the mastectomy skin flap. Seromas in this location are also characterized by the gradual formation of a thin capsule that forms over the subcutaneous fat that may impede lymphatic flow resulting in edema and rubor. The use of perforated or fenestrated ADM has been postulated to allow for egress of fluid between the skin and ADM and thus decrease the incidence of early seroma formation (26-29).

**Evidence for ADM Revascularization**

It is important to recognize that unlike a skin graft where inosculation occurs between days 2 – 5, the revascularization of an ADM has a more protracted course. The evidence for this is derived from experimental studies looking specifically at this. In a murine study evaluating ADM revascularization, it was demonstrated that increased oxygen consumption and angiogenesis along the edges of the ADM occurs from days 10 to 14 with vascular and inflammatory cell penetration into the center of the ADM after about 21 days (30). In another experimental study using a porcine model, it was demonstrated that early angiogenesis occurs at 4 weeks at the interface of the ADM and skin flap (31). The revascularization of both surfaces of the ADM is evident by 8 weeks (Figure 2). Microcirculatory evaluation using a video microscope demonstrated detectable flow 12 weeks after implantation. These histological changes related to angiogenesis may serve as a foundation for the self-limiting nature of RBS.

**ADM Adherence versus Revascularization**

It is important to differentiate ADM adherence from revascularization because the two are distinctly different. Adherence alone, due to scar formation, does not imply revascularization or recellularization and will not provide any physiological function or benefit. Clinical evidence for revascularization is based on a pink hue, vascularized hair follicles, and actual vascular ingrowth (Figure 2). In order for lymphatic function within ADM to occur, revascularization and recellularization of ADM is required. Lymphatic function will be compromised in the presence of dense scar; therefore, it is important to use an ADM that will confer regenerative potential rather than a scarred scaffold. It is important for clinicians to be aware of the differences in ADM performance and select accordingly.

**Lymphatic Obstruction and Red Breast Syndrome**

When the normal path of lymphatic flow is obstructed or the mechanical forces promoting lymphatic flow are disrupted, erythema and edema of the mastectomy skin flaps may ensue (32). This may be the result of various inflammatory mediators that in some cases may be associated with mild bacterial overgrowth (32). Early resolution of RBS is most likely the result of lymphangiogenesis within the mastectomy skin flaps and ADM that will gradually result in a diminution of the erythema. This process may be expedited by ADMs that revascularize and recellularize relatively quickly. Late resolution of or persistent RBS is postulated to be due to the absence of lymphatic connections within the mastectomy skin flap and may be amplified by an ADM that has failed to revascularize or recellularize, thus never permitting the normal drainage of the cutaneous lymphatic system.

**Infection versus Red Breast Syndrome**

The differentiation between RBS and infection is important because both can occur within a similar timeframe and have similar clinical characteristics; however, the two have a distinctly different pathophysiology (1,3). Red breast syndrome is usually self-limiting and will resolve without treatment; whereas, infection is usually progressive and will cause deterioration and reconstructive failure over time if untreated (figures 3-4). External cues that can assist in differentiating the two include the extent or location of the erythema. The erythema associated with RBS is usually over the ADM, whereas the erythema associated with infection may extend beyond the borders of the ADM. When the erythema extends outside the territory of the ADM, superficial or deep infection must be considered (figure 5). In some cases of protracted RBS, the erythema can extend beyond the borders of the ADM (figure 6).

When breast erythema is noted following mastectomy and ADM use, the initial response should be to rule out infection. The incidence of surgical site infection in the setting of ADM ranged from 0 – 18.5% with a mean incidence of 5.5% in the nine studies reviewed (table 1). The initial evaluation requires a through history and physical examination and obtaining appropriate laboratory studies. Prior investigation has demonstrated that the primary hallmarks of infection are pain, redness and swelling and that fever and leukocytosis are less common (33). In patients with RBS, fever, leukocytosis and pain are uncommon; however, erythema is universal with or without breast pain or edema. The onset and duration of RBS is variable ranging from a few days to a few weeks and a few weeks to several months respectively. The self-limiting aspect of RBS is postulated to be the result of angiolymphatic regeneration and the re-establishment of lymphatic flow thereby resulting in the resolution of the inflammatory mediators responsible for the localized erythema.

In all cases of erythema, a trial of antibiotic therapy is recommended that may be administered orally if mild or intravenously if severe. Cellulitis will typically resolve with antibiotics or require operative exploration if progressive. Red breast syndrome will usually be unaffected with antibiotic therapy but can progress to infection if there is a component of bacterial overgrowth. If there is no change after one week of therapy and the patient remains afebrile, RBS is presumed and the antibiotics are discontinued. Figures 6 and 7 illustrate a patient with RBS of 9 months duration who eventually had explantation of the original implants and ADM followed by secondary reconstruction. The decision to convert from prosthetic to autologous reconstruction in the setting of protracted RBS is based on the quality of the reconstruction, patient concerns and surgeon recommendation.

**Understanding ADM sterility**

Several retrospective clinical studies have reviewed the incidence of RBS and attempted to determine its etiology without any physiologic explanations (Table 1). In one study comparing aseptic AlloDerm to sterile AlloDerm, it was demonstrated that the incidence of RBS decreased from 7.5% to 2.5% (3). In another study, the incidence of surgical site infection was 11.1% with aseptic and 7.7% with sterile AlloDerm (24). The difference between aseptic and sterile AlloDerm is that the aseptic AlloDerm is freeze dried and has a sterility assurance level (SAL) of 10^-3^, whereas sterile AlloDerm is terminally sterilized using radiation and has a SAL that is also 10^-3^. Given that the incidence of RBS was reduced but not eliminated with the sterile product, the authors recognized that the occurrence of RBS might be unrelated to the processing of ADM. Although other ADMs were not evaluated, the study implied that RBS could occur with any ADM. In another study using a mathematical model to evaluate the relationship of SAL to infection, it was demonstrated that there was no difference in the rate of infection when comparing ADM with a SAL of 10^-3^ and 10^-6^ (34).

The purpose of device or tissue sterilization is to reduce the bacterial count. Guidelines for sterilization set forth by the FDA are that for a product to be considered sterile, a minimal SAL of 10^-3^ must be achieved using terminal sterilization techniques such as radiation or detergents. A SAL of 10^-3^ implies that the likelihood of finding a viable organism is one in a thousand whereas a SAL of 10^-6^ would be one in a million. Standards for the sterilization of medical devices or tissues will depend on the nature of the material. Materials that are heat resistant such as metals are best sterilized to an SAL ranging from 10^-6^ – 10^-9^. This is in contrast to materials that are heat sensitive that are typically sterilized to an SAL of 10^-3^. (35) Human acellular dermal matrices are thermally sensitive tissues that can be damaged by excessive radiation. The implantation of a damaged human ADM is far more likely to result in an inflammatory reaction as the body undergoes degradation processes to eradicate the material from the body. It is postulated that the refractory nature of the RBS may be the result of scarred interface between the ADM and the mastectomy skin flap compromising the flow of lymphatic fluid and resulting in protracted RBS.

**Clinical studies**

There have been several comparative clinical outcome studies evaluating various ADM materials. In one publication comparing dual plane reconstruction using AlloDerm (Allergan Inc. Irvine, CA) to DermaCell (Stryker, Kalamazoo, MI), the authors concluded that RBS was increased with AlloDerm (26%) compared to Dermacell (0%). (10) The primary explanation for this observation was that Dermacell was sterilized to a SAL of 10^-6^ whereas AlloDerm is sterilized to a SAL of 10^-3^. The authors concluded that RBS is an inflammatory response to ADM and that by aggressive sterilization of ADM, RBS would be eliminated. Their conclusion that RBS is due to the degree of ADM sterilization is not based on any physiologic explanation and represents conjecture. Their contention is that RBS is inflammatory is accurate; however, the implication that it is minimized by increasing the sterility assurance level to 10^-6^ is without foundation and misrepresentative.

It is important to recognize that RBS is not product specific. It can occur with any ADM regardless of the degree of sterilization or the biologic source (Table 1). This has been demonstrated by the clinical studies as well as personal observation having used a variety of ADMs and having evaluated patients with RBS that have had different ADMs placed (Table 1). It is also important to appreciate that RBS is uncommon with a mean occurrence based on review of the 8 studies of 6.4%.

**Conclusion**

In conclusion, RBS is more likely to represent the rubor associated with lymphedema and lymphatic obstruction rather than the type of ADM used or the other possible etiologies mentioned. It is important to recognize that these conclusions are based on the best available evidence and is not intended to be absolute. Inflammation is multifactorial, but the clinical appearance and characteristics of RBS is constant. Understanding the possible mechanisms responsible for RBS is important as we move forward with prosthetic breast reconstruction and ADM.

**Reference**

1. Wu PS, Winocour S, Jacobson SR. Red Breast Syndrome: A review f the available literature. Aesth Plast Surg. 39: 227-230, 2015.
2. Nahabedian M, Cocilovo C. Two-Stage Prosthetic Breast Reconstruction: A Comparison Between Prepectoral and Partial Subpectoral Techniques. Plast. Reconstr. Surg. 140: 22S, 2017.
3. Lewis P, Jewell J, Mattison G, Gupta S, Kim H. Reducing postoperative infections and red breast syndrome in patients with acellular dermal matrix-based breast reconstruction: the relative roles of product sterility and lower body mass index. Ann Plast Surg. 74 Suppl 1:S30-2, 2015.
4. Govshievich A, Somogyi RB, Brown MH. Conservative mastectomies and immediate reconstruction with the use of ADMs. Gland Surg. 4(6): 453-462, 2015.
5. Eichler C, Vogt N, Brunnert K, et al. A Head-to-head Comparison between Surgimend and Epiflex in 127 Breast Reconstructions. Plast Reconstr Surg Glob Open. 3(6): e439, 2015.
6. Eichler C, Efremova J, Brunnert K, et al. A head to head comparison between Surgimend fetal bovine acellular dermal matrix and tutomesh a bovine pericardium collagen membrane in breast reconstruction in 45 cases. In Vivo. 31:677 – 682, 2017.
7. Momeni A, Kanchwala S. Improved pocket control in immediate microsurgical breast reconstruction with simultaneous implant placement through the use of mesh. Microsurgery. 38: 450-457, 2018.
8. Ortiz JA. Clinical Outcomes in Breast Reconstruction Patients Using a Sterile Acellular Dermal Matrix Allograft. Aesthetic Plastic Surgery. 41(3); 542–550, 2017
9. Venturi ML, Mesbahi AN, Boehmler JH IV, et al. Evaluating sterile human acellular dermal matrix in immediate expander-based breast reconstruction: A multicenter, prospective, cohort study. Plast Reconstr Surg. 131:9e–18e, 2013.
10. Pittman T, Fan K, Knapp A, Frantz S. Spear S. Comparison of Different Acellular Dermal Matrices in Breast Reconstruction: The 50/50 Study. Plast. Reconstr. Surg. 139: 521, 2017
11. Nahabedian MY. AlloDerm Performance in the Setting of Prosthetic Breast Surgery, Infection, and Irradiation. Plast. Reconstr. Surg. 124: 1743, 2009.
12. Newman MI, Hanabergh E, Samson MC. (Reply) AlloDerm Performance in the Setting of Prosthetic Breast Surgery, Infection, and Irradiation Plast Reconstr Surg. 126(3): 1120, 2010.
13. Nahabedian MY (reply). (Reply) AlloDerm Performance in the Setting of Prosthetic Breast Surgery, Infection, and Irradiation Plast Reconstr Surg. 126(3): 1120-1, 2010.
14. Ganske I, Hoyler M, Fox SE, et al. Delayed hypersensitivity reaction to acellular dermal matrix in breast reconstruction: the red breast syndrome? Ann Plast Surg. 73:S139–S143, 2014.
15. Suami H, Pan WR, Taylor GI. Historical review of breast lymphatic studies. Clin Anat 22(5): 531-6, 2009.
16. Suami H, Pan WR, Mann B, Taylor GI. The Lymphatic Anatomy of the Breast and its Implications for Sentinel Lymph Node Biopsy: A Human Cadaver Study. Ann Surg Oncol. 15(3): 863-71, 2008.
17. Tanis PJ, Niewig OE, Valdes Olmos RA, Broon BR. Anatomy and Physiology of Lymphatic Drainage of the Breast from the Perspective of Sentinel Node Biopsy. J Am Coll Surg. 193(3): 399-409, 2001
18. Cichowitz A, Stanley PA, Morrison WA. Erysipelas-like inflammation following breast surgery. J Plast Reconstr Aesthet Surg. 60(5): 490-4, 2007.
19. Zetterlund L, Axelsson R, Svensson L, Perbeck L, Celebioglu F. Lymphatic drainage in the breast before and up to five years after a reduction mammaplasty. Lymphology. 49(3): 157-64, 2016.
20. Campbell KT, Burns NK, Ensor J, Butler CE. Metrics of Cellular and Vascular Infiltration of Human Acellular Dermal Matrix in Ventral Hernia Repairs. Plast. Reconstr. Surg. 129: 888, 2012.
21. Heyer K, Buck DW, Kato C, et al. Reversed acellular dermis: failure of graft incorporation in primary tissue expander reconstruction resulting in recurrent breast cellulitis. Plast Reconstr Surg. 125(2): 66e-68e, 2010.
22. Ollech CJ, Block LM, Afifi AM, Poore SO. Effect of Drain Placement on Infection, Seroma, and Return to Operating Room in Expander-Based Breast Reconstruction. Ann Plast Surg. 79(6): 536-540, 2017.
23. Jordan SW, Khavanin N, Kim JYS. Seroma in prosthetic breast reconstruction. Plast. Reconstr. Surg. 137: 1104, 2016.
24. Hanson SE, Meaike JD, Selber JC, et al. Aseptic Freeze-Dried versus Sterile Wet-Packaged Human Cadaveric Acellular Dermal Matrix in Immediate Tissue Expander Breast Reconstruction: A Propensity Score Analysis. Plast. Reconstr. Surg. 141: 624e, 2018
25. Heidemann LN, Gunnarson GL, Salzberg CA, Sorensen JA, Thomasen JB. Complications following Nipple-Sparing Mastectomy and Immediate Acellular Dermal Matrix Implant-based Breast Reconstruction—A Systematic Review and Meta-analysis. Plast Reconstr Surg Glob Open. 6:e1625, 2018.
26. Paydar K, Wirth GA, Mowlds DS. Prepectoral Breast Reconstruction with Fenestrated Acellular Dermal Matrix: A Novel Design. Plast Reconstr Surg Glob Open. 6:e1712, 2018.
27. Martin JB, Moore R, Paydar KZ, Wirth GA. Use of fenestrations in acellular dermal allograft in two-stage tissue expander/implant breast reconstruction. Plast Reconstr Surg. 134(5): 901-904, 2014.
28. Palaia DA, Arthur KS, Cahan AC, Rosenberg MH. Incidence of Seromas and Infections Using Fenestrated versus Nonfenestrated Acellular Dermal Matrix in Breast Reconstructions. Plast Reconstr Surg Glob Open. 3(11): e569, 2015.
29. Frey JD, Alperovich M, Weichman KE, et al. Breast Reconstruction Using Contour Fenestrated AlloDerm: Does Improvement in Design Translate to Improved Outcomes? Plast Reconstr Surg Glob Open 3:e505, 2015.
30. DeGeorge BR, Ning B, Salopek LS, et al. Advanced Imaging Techniques for Investigation of Acellular Dermal Matrix Biointegration. Plast Reconstr Surg. 139(2): 395-405, 2017.
31. Garcia O, Scott JR. Analysis of acellular dermal matrix integration and revascularization following tissue expander breast reconstruction in a clinically relevant large-animal model. Plast Reconstr Surg. 131(5): 741-751, 2013.
32. Von Der Weid PY. Review article: lymphatic vessel pumping and inflammation - the role of spontaneous constrictions and underlying electrical pacemaker potentials. Aliment Pharmacol Ther. 15: 1115-1129, 2001.
33. Nahabedian MY, Tsangaris T, Momen B, and Manson PN. Infectious Complications Following Breast Reconstruction with Expanders and Implants. Plast Reconstr Surg 112: 467-476, 2003.
34. Srun SW, Nissen BJ, Bryans TD, Bonjean M. Medical Device SALs And Surgical Site Infections: A Mathematical Model. Biomed Instrument Tech. May/June: 230-238, 2012
35. Von Woedtke T, Kramer A. The limits of sterility assurance. GMS Krankenhaushygiene Interdisziplinär. 3(3): 1-10, 2008.

**Legends**

Table 1. Clinical outcomes from 9 studies comparing the incidence of surgical site infection (SSI), red breast syndrome (RBS) and seroma. AE – adverse event, BMI – body mass index, FU – follow-up, NR – not reported

Table 2. The various etiologies of red breast syndrome are listed with the corresponding study.

Table 3. Truths and myths of red breast syndrome are highlighted.

Figure 1. Red breast syndrome in a patient following prepectoral breast reconstruction with ADM.

Figure 2. A pink hue, vascularized follicles and the demonstration of small vascular channels characterize revascularized ADM.

Figure 3. Red breast syndrome can mimic the appearance of cellulitis

Figure 4. Cellulitis can mimic the appearance of red breast syndrome.

Figure 5. An example of erythema in a patient with dual plane breast reconstruction. The redness extends outside the borders of the ADM characteristic of cellulitis.

Figure 6. Following bilateral breast reconstruction with tissue expanders and ADM, The left implant was removed secondary to infection. The right breast was erythematous for 9 months with protracted inflammation due to red breast syndrome.

Figure 7. Following explantation and bilateral breast reconstruction with latissimus dorsi musculocutaneous flaps and implants.

SDC1 – See video, Supplemental Digital Content 1 which displays pitting edema demonstrated in a patient with red breast syndrome. INSERT LINK HERE
